# Metabolic and Phytotoxic Profile of Phytopathogens in Main Extensive Crops of Argentina

**DOI:** 10.3390/toxins17090466

**Published:** 2025-09-18

**Authors:** Francisco José Sautua, Maria Chiara Zonno, Pierluigi Reveglia, Maria Letizia Ciavatta, Marianna Carbone, Lucia Lecce, María Cecilia Pérez-Pizá, Gaetano Corso, Marcelo Anibal Carmona, Antonio Evidente

**Affiliations:** 1 Cátedra de Fitopatología, Facultad de Agronomía, Universidad de Buenos Aires, Av. San Martin 4453, Buenos Aires 1417, Argentina; mperez@agro.uba.ar (M.C.P.-P.); carmonam@agro.uba.ar (M.A.C.); 2 Institute of Sciences of Food Production, National Research Council, Via Amendola 122/O, 70125 Bari, Italy; maria.zonno@cnr.it; 3 Department of Clinical and Experimental Medicine, University of Foggia, Via Gramsci, 89/91, 71121 Foggia, Italy; lucia.lecce@unifg.it (L.L.); gaetano.corso@unifg.it (G.C.); 4 Institute of Biomolecular Chemistry, National Research Council, Viale Campi Flegrei 34, 80078 Pozzuoli, Italy; marialetizia.ciavatta@cnr.it (M.L.C.); marianna.carbone@cnr.it (M.C.); evidente@unina.it (A.E.); 5 Instituto de Biología Funcional y Biotecnología, Instituto de Investigaciones en Biodiversidad y Biotecnología, Consejo Nacional de Investigaciones Científicas y Técnicas, Vieytes 3103, Buenos Aires 7600, Argentina

**Keywords:** Argentine crops, phytopathogenic fungi, phytotoxic activity, phytotoxins, LC-MS/MS

## Abstract

Phytopathogenic fungi represent a significant biotic stress affecting global agriculture, often causing severe diseases and, in some cases, leading to plant death. They have been isolated from economically important crops, including cereals, legumes, and fruits. Among the compounds produced by fungi, phytotoxins play a key role in disease development by interfering with host physiological processes. In this study, organic extracts from *Cercospora kikuchii*, *Cercospora nicotianae*, *Cercospora sojina*, *Diaporthe longicolla*, *Septoria glycines*, *Pyrenophora teres*, and *Pyrenophora tritici-repentis*, isolated from three major Argentine crops, were first screened for the in vitro production of phytotoxic metabolites. Subsequently, selected metabolites were dereplicated using liquid chromatography-tandem mass spectrometry (LC-MS/MS) and nuclear magnetic resonance (NMR) spectroscopy. The phytotoxins identified varied according to the fungal species and extraction conditions. Cercosporin, putaminoxin, scytalone, and isosclerone were identified. These findings underscore the need for further chemical investigation to comprehensively characterize the metabolome of these phytopathogens and clarify their roles in plant–pathogen interactions.

## 1. Introduction

Some of the most fertile agricultural lands in Argentina are located in the Pampa Húmeda (Humid Pampas), a region that covers the central and northeastern regions of the country. This area comprises major agricultural hubs where diverse crops are grown, including soybeans, wheat, corn, sunflowers, barley, peas, canola, grass seeds, sorghum, and fodder [1,2].

As in agricultural systems worldwide, these crops are subject to yield reductions caused by biotic and abiotic stresses. Abiotic factors, including human-induced environmental pollution, contribute substantially to yield losses [3,4]. This issue is especially concerning in light of global demographic trends: the global population is projected to reach nearly 10 billion by 2050 [5,6], placing increasing pressure on food security.

Among biotic stresses, microbial diseases pose a critical threat, with phytopathogenic fungi being major contributors to crop losses. These pathogens reduce agricultural productivity and compromise food safety [7]. In Argentina, recent studies have focused on fungal pathogens such as *Macrophomina phaseolina* and *Colletotrichum truncatum*, which have been investigated through collaborations between plant pathologists and natural product chemists, including members of the present research team [8,9]. Additionally, several other phytopathogenic fungi exert substantial economic and social impacts on major crops in the country. These include species of *Cercospora* (e.g., *C. kikuchii*, *C. sojina*, *C. nicotianae*), *Diaporthe* (e.g., *D. longicolla*), *Septoria* (e.g., *S. glycines*), and *Pyrenophora* (e.g., *P. teres* and *P. tritici-repentis*). In Argentina, *Cercospora* and *Pyrenophora* species can cause significant yield losses in soybean and cereal crops, respectively [10,11,12].

Therefore, control of pathogenic fungi in agriculture is increasingly necessary. Management strategies in crop production include mechanical, cultural, chemical, and biological approaches, with chemical control playing a predominant role. However, the emergence of resistance to several fungicide active ingredients has limited the effectiveness of this approach, meaning that current strategies have not fully addressed the challenges posed by fungal diseases [10,11,12,13,14]. Consequently, considerable efforts have focused on developing alternative strategies based on natural products, particularly those involving microbial- or plant-derived metabolites, applied either individually or in combination as part of more efficient and integrated management systems [14,15,16,17,18,19,20].

Nevertheless, research primarily focused on the plant perspective provides only a partial view and often lacks a comprehensive understanding of host–pathogen interactions. Fungi produce a wide range of metabolites, including specialized metabolites that damage crops and play central roles in the phytopathogenic process [21]. These metabolites must be considered to fully elucidate fungal pathogenesis. Phytotoxins, fundamental in triggering disease symptoms, encompass diverse classes of natural compounds with varied biological activities [22,23,24,25]. Investigating the chemical space of phytopathogenic fungi is therefore essential to determine whether the compounds produced in vitro are also synthesized under in planta conditions.

Fungal growth conditions strongly influence phytotoxin production in vitro [26]. Culture medium and extraction method significantly affect the type and quantity of specialized metabolites recovered, thereby shaping the chemical profile of the resulting extracts. Given this complexity, in-depth studies are often required, frequently applying the “One Strain, Many Compounds” (OSMAC) approach [27], together with optimized extraction and purification methods, to explore the full metabolic potential of phytopathogenic fungi. However, fungi often produce previously known compounds, which are sometimes only identified after labor-intensive and costly purification processes. To address this challenge, rapid and accurate dereplication of known compounds is now a crucial step in natural product research [28]. Traditional approaches, such as compound isolation followed by structural elucidation using nuclear magnetic resonance (NMR), remain valuable but are costly and generally feasible only for high-priority candidates with established or potential bioactivity. Thus, early differentiation between known and novel compounds is essential to accelerate the discovery of bioactive metabolites and avoid redundant efforts. Dereplication not only streamlines the discovery pipeline but also helps prioritize structurally novel molecules, thereby optimizing human and financial resources [28]. Tandem mass spectrometry (MS/MS) has emerged as a powerful dereplication strategy by providing fragmentation patterns that can be matched to public spectral databases, including those specialized in microbial natural products such as NPAtlas [29,30]. In addition, both targeted and untargeted metabolomics have proven invaluable for profiling the chemical diversity of fungal metabolites [31,32].

In this context, this preliminary study screened organic extracts from *C. kikuchii*, *C. nicotianae*, *C. sojina*, *D.e longicolla*, *S. glycines*, *P. teres*, and *P. tritici-repentis*, isolated from three major Argentine crops (soybean, wheat, and barley), for the in vitro production of phytotoxic metabolites. Dereplication was conducted applying liquid chromatography-tandem mass spectrometry (LC-MS/MS) and NMR spectroscopy on fungal culture filtrates.

## 2. Results and Discussion

Soybean, wheat, and barley are among the main large-scale grain crops cultivated annually in Argentina. Several fungal pathogens are responsible for endemic diseases in the Pampas region, such as leaf spots (*C. sojina*, *S. glycines*, *P. teres*, *P. tritici-repentis*), leaf blights (*C. kikuchii*, *C. nicotianae*), seed decay, and pod and stem blight (*D. longicolla*), which occur annually and cause substantial economic losses for producers [10,11,12]. For the present study, a set of fungal isolates representing these pathogens was obtained from the fungal culture collection of the Plant Pathology Department, Faculty of Agronomy, University of Buenos Aires (see Section 4.2. Fungi) and grown in liquid culture to enable dereplication of phytotoxins in the organic extracts and to evaluate their phytotoxicity on target plants.

### 2.1. Extraction of Culture Filtrates and Phytoxic Assay

All fungal isolates were able to produce metabolites, and extract yields at the original pH of the culture filtrate and at pH 2 were generally similar, except for *C. kikuchii*, whose yield was much lower at pH 2 (1.5 mg), as shown in Table 1. Total yields ranged from 12.4 mg for *C. kikuchii* at the original pH to 186.4 mg for *P. teres* at pH 2.

To evaluate the phytotoxic potential of the organic extracts from the fungal species listed in Table 1, a preliminary leaf puncture bioassay was conducted on *Vigna* sp. (mung bean), *Solanum lycopersicum* L. cv. Regina di Fasano (tomato), and *Glycine max* Merr. (soybean). The extracts were tested at both their original pH and under acidic conditions (pH 2) to assess the influence of pH on phytotoxic activity. All extracts exhibited phytotoxic effects across the tested plant species. These results are summarized in Table 2.

Among the tested extracts, *P. teres* demonstrated the highest phytotoxicity, inducing necrotic lesions exceeding 8 mm in diameter. *C. kikuchii* exhibited moderate phytotoxic activity at its original pH, while *C. nicotianae* showed a similar level of toxicity but only under acidic conditions (pH 2). *P. tritici-repentis* displayed a comparable phytotoxic effect. The remaining extracts induced only low necrotic symptoms under all tested conditions.

The observed phytotoxicity, particularly for species within the *Cercospora and Pyrenophora* genera, likely reflects their known capacity to produce broad-spectrum specialized metabolites with phytotoxic, antibacterial, and antifungal properties [25]. These findings support the progression to the next phase of the study, which involves a chemical investigation and targeted dereplication of specialized fungal metabolites to identify the bioactive compounds responsible for the observed effects.

### 2.2. Preliminary TLC Analysis and Optimization of Targeted LC-MS/MS Analysis

TLC was used for the preliminary analysis of fungal organic extracts. The chromatographic profiles revealed the presence of several secondary metabolites with different polarities in *C. kikuchii*, *D. longicolla*, *P. teres*, and *P. tritici-repentis*, whereas *C. Sojina* and *S. glycines* produced fewer metabolites. Although TLC provides an initial indication of metabolic diversity, it captures only a limited portion of the complex chemical diversity of phytopathogenic fungi. Building on these results, future studies will apply non-targeted metabolomics in combination with the OSMAC approach. This strategy, coupled with advanced data analysis workflows, can reveal differences in metabolic fingerprints across fungal species or growth conditions that may be overlooked by TLC. Nevertheless, non-targeted metabolomics also presents limitations, as it often allows only putative metabolite identification and may not distinguish between stereoisomers. For this reason, dereplication strategies involving authentic standards remain essential to confirm metabolite identities and to discriminate between already known or common metabolites produced by the selected fungal species. To enable the rapid dereplication of phytotoxic metabolites present in organic extracts from the selected pathogenic fungi, a targeted LC-MS/MS method was developed. A library of 23 pure metabolites previously isolated from pathogenic fungi and unambiguously characterized by NMR spectroscopy was assembled. Supplementary Table S1 shows the chemical structures, fungal sources, and corresponding literature for each toxin used as a reference standard. These compounds were used to construct a customized in-house database with optimized multiple reaction monitoring (MRM) parameters. This strategy eliminates the need for labor-intensive chromatographic purification for dereplication, significantly reducing the use of organic solvents and making the process more efficient and environmentally sustainable. Nevertheless, it is essential to acknowledge that this strategy also has limitations; indeed, the reliability of dereplication depends heavily on the comprehensiveness of spectral databases, which are typically incomplete, especially for microorganisms, and underrepresent many rare or undiscovered fungal metabolites. Finally, fungal metabolomes are highly dynamic, with specialized metabolites’ expression influenced by culture conditions, making dereplication insufficient to capture the full chemical diversity. The complete list of optimized metabolites, along with their corresponding mass spectrometric parameters, is presented in Table 3.

**Figure 1:**
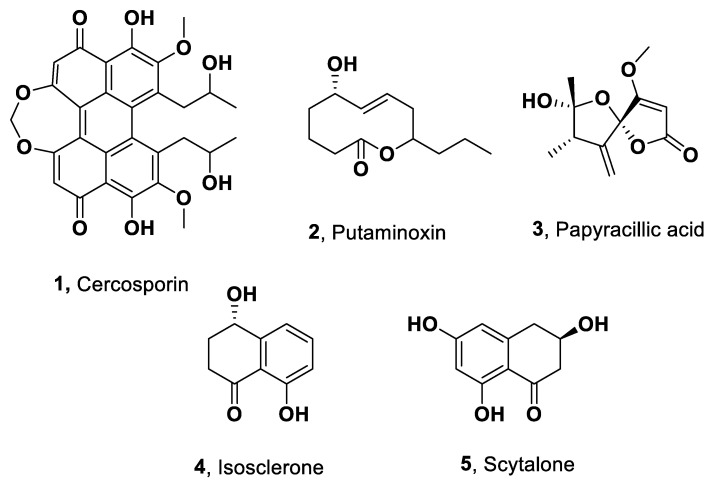
Structure of phytotoxins identified in the organic extract of selected culture filtrates analyzed by LC-MS/MS.

The metabolites identified in the extracts of the selected fungal species are summarized in Table 4, and their structures are shown in Figure 1.

Our results further emphasize that the application of mass spectrometry in the dereplication of specialized metabolites, particularly in the context of metabolomics, has been transformative. Rapid dereplication using tandem MS, especially when combined with database comparison (either online or in-house), represents a major advance in natural product discovery, as it substantially reduces time and resource demands. Recent progress in analytical chemistry and bioinformatics, particularly the advent of molecular networking, have revolutionized natural product research [32]. Molecular networking enables the rapid organization and visualization of MS/MS datasets, automating annotation through database comparison and facilitating the identification of novel compounds within complex mixtures. A key limitation of this approach, however, is its reliance on putative identifications, as definitive confirmation requires pure reference standards, which are often unavailable, especially for rare or unique fungal metabolites. More recently a comprehensive method known as biochemometrics has emerged as a powerful tool for the targeted discovery of bioactive constituents by correlating chemical profiles with biological activity data. Integrating MS and NMR workflows enhances both confidence in metabolite annotation and the efficiency of natural product discovery [33,34].

### 2.3. Metabolites from C. nicotinae

The case of *C. nicotianae* was particularly noteworthy, as it exclusively produced a single, homogeneous metabolite: cercosporin (**1**, Figure 1). This dihydroxy-perylenequinone was first described in 1957 as a red pigment isolated in high yields from *C. kikuchii* (syn. *Cercosporina kikuchii*), a fungal pathogen responsible for purple seed stain in soybean [35]. The initial study reported its molecular formula and key functional groups, although its complete structure was not yet elucidated. The structure of cercosporin was later revised and definitively determined through spectroscopic analysis [36], and its absolute stereochemistry was subsequently established using optical methods [37].

In the present study, cercosporin (**1**) was not detected in either *C. kikuchii* or *C. sojina*. Instead, it was identified as the sole metabolite in the organic extract of *C. nicotianae*, consistent with previous reports for this species [38]. Structural confirmation of cercosporin was achieved by comparing its spectroscopic and physicochemical properties, including NMR and MS spectra provided in Supplementary Materials (Figures S1–S4), with those previously described in the literature [36,37]. Cercosporin has been recognized as a major phytotoxin produced by several *Cercospora* species responsible for economically significant leaf spot and blight diseases worldwide [39]. These include Cercospora leaf spot of sugar beet (*C. beticola*), grey leaf spot of maize (*C. zeae-maydis*), purple seed stain of soybean (*C. kikuchii*), frog eye leaf spot of tobacco (*C. nicotianae*), and brown eye spot of coffee (*C. coffeicola*). Under the preliminary in vitro conditions used in this study, the Argentine strain of *C. kikuchii* produced only small amounts of metabolites, as shown by TLC analysis of the culture extract. Notably, cercosporin isolated from *C. nicotinae* (see above) had already been identified prior to the LC-MS/MS analyses of all fungal organic extracts and was therefore not used as a reference standard. Cercosporin is the only fungal toxin classified as a photosensitizer. It belongs to a broader class of natural compounds that are activated by visible light, which generate reactive oxygen species (ROS) through a mechanism known as photodynamic action. The ROS produced damage plant cell membranes, thereby facilitating the release of nutrients that support the growth of these intercellular fungal pathogens [40].

### 2.4. Metabolites from C. kikuchii and C. sojina

*C. kikuchii* and *C. sojina* were found to produce putaminoxin (**2**, Figure 1) at pH 2 and 9, as reported in the LC-MS/MS library (Table 3 and Figure S5A,B in Supplementary Materials). Putaminoxin is the main phytotoxin produced by *Phoma putaminum*, the causal agent of leaf necrosis of *Erigeron annuus*, a common weed of field and pasture [25]. Compound **2**, produced together with several analogues (putaminoxin B-E) from the same fungus, belongs to the macrolide class of natural products, specifically to the subgroup of nonenolides. When tested on leaves of host and non-host plants, putaminoxin (**2**) showed a wide range of toxicity, with *E. annuus* being the most sensitive [25]. Other phytotoxic nonenolides have also been reported from fungi pathogenic to crops (e.g., pinolidoxins) and weeds (e.g., herbarumin and stagonolides), such *as Dymidella pinodes*, *Phoma herbarum*, and *Stagonospora cirsii*, respectively. These nonenolides and some of their semisynthetic derivatives have been extensively investigated in structure–activity relationship studies [25,41]. However, the present study constitutes the first report of putaminoxin production by *C. kikuchii* and *C. sojina*. The production of this metabolite by soybean pathogens is not unexpected, as pinolidoxin has been identified as the main phytotoxin produced by *D. pinodes*, a pathogen of peas [25].

### 2.5. Metabolites from D. longicolla

*D. longicolla* was found to produce papyracillic acid (**3**, Figure 1) and isosclerone (**4**, Figure 1) at pH 5 and 2, respectively (Table 3 and Figure S5C,D in Supplementary Materials). Compound 3 was first isolated as a phytotoxin from *Ascochyta agropyrina* var. *nana*, a pathogen proposed as a mycoherbicide against *Elytrigia repens* (quack grass). This plant is a noxious perennial weed that is widespread throughout the cold regions of both the Northern and Southern hemispheres. Tested by leaf disk-puncture assay, papyracillic acid (**3**) showed phytotoxic activity on both the host plant and several nonhost plants. Compound **3** was also active against bacteria such as *Xanthomonas campestris* and *Bacillus subtilis*, as well as the fungus *Candida tropicalis* [42]. This is in agreement with the high antimicrobial, nematicidal, and cytotoxic activity of compound **3** previously reported when it was isolated from the ascomycete *Lachnum papyraceum* [43,44]. Isosclerone (**4**, Figure 1) is a pentaketide naphthalenone, reported together with scytalone (**5**, Figure 1) as a phytotoxin produced by *Phaeoacremonium aleophilum* and *Phaeomoniella chlamydospora*, two fungi involved in grapevine Esca disease. Isosclerone and scytalone (**4** and **5**), when tested on detached leaves of grapevine, caused large, coalescent chlorotic and necrotic spots followed by distortion of the lamina and withering and light green to chlorotic, rounded to irregular, interveinal or marginal spots, respectively [25]. Isosclerone (**4**) has been found, in both enantiomeric forms, as a secondary metabolite of several fungi and plants, while its (-)-enantiomer is called regiolone [25]. The absolute configurations of regiolone and isosclerone, produced by *Botrytis cinerea* and *Botrytis fabae*, respectively, were unambiguously assigned by ab initio computational prediction of their theoretical optical rotatory powers and electronic circular dichroism spectra. The (*R*) configuration at C-4 was found to be a fun-damental structural feature for phytotoxicity, as demonstrated by the activity of the two compounds tested for comparison on faba bean (host of both pathogens) and grapevine (host of *B. cinerea* only) [25]. Phytotoxin **4** is also produced by *Neofusicoccum parvum*, a Botryosphaeriaceae pathogen of grapevine, together with (3*R*,4*R*)-(-)-4-hydroxymellein, (3*R*,4*S*)-(-)-4-hydroxymellein, and tyrosol. When assayed for phytotoxicity on tomato plants, all four metabolites showed phytotoxic activity, with (3*R*,4*R*)-(-)-4-hydroxymellein and isosclerone as the most active compounds [25]. Finally, isosclerone was isolated together with pyriculins A and B, two monosubstituted hex-4-ene-2,3-diols, (10*S*,11*S*)-(-)-epipyriculol, *tran*s-3,4-dihydro-3,4,8-trihydroxy-1(2*H*)-napthalenone from *Pyricularia grisea*. This fungus is a foliar pathogen of buffelgrass (*Cenchrus ciliaris*) in North America, where the species is considered an invasive weed, and has therefore been studied as a potential biocontrol agent. All the isolated metabolites delayed germination, but only (10*S*,11*S*)-(-)-epipyriculol was able to prevent radicle development in buffelgrass seedlings while not affecting coleoptile elongation [45].

### 2.6. Metabolites from S. glycines, P. teres, and P. tritici-repentis

The other three fungi studied (*S. glycines*, *P. teres*, and *P. tritici-repentis*) were found to produce scytalone (**5**), as identified by LC-MS/MS (Table 4 and Figure S5E–G in Supplementary Materials). Scytalone is a precursor of melanin, the black pigment produced by the brown brm-1 mutant of *Verticillium dahliae*. Related metabolites such as flaviolin and *cis*-4-hydroxyscytalone were also isolated. Flaviolin and its derivatives contribute to the reddish-brown color of the brm-1 mutant. Scytalone plays a key role in the biosynthetic pathway of melanin in fungi [46,47]. In contrast, juglone, 2-hydroxyjuglone, 3-hydroxyjuglone, and *cis*-(-)-3,4-dihydro-3,4,8-trihydroxy-1- (2*H*)-naphthalenone were isolated from the culture filtrates of the melanin-deficient brm-2 mutant of *V. dahliae*. Compound **5** is also a phytotoxic pentaketide naphthalenone produced by several pathogens of agricultural crops, particularly those affecting grapevine [48,49]. Phytotoxin **5** was also isolated together with isosclerone (**4**) from several *Phaeoacremonium* species pathogenic to grapevines, such as *P. italicum*, *P. alvesii*, and *P. rubrigenum*, which induce diverse disease symptoms in the host plants [50]. Scytalone (**5**) was also produced by *Lasiodiplodia theobromae*, a phytopathogenic fungus associated with many host plants, inducing different and severe diseases on crops such as mango and grapevines. This fungus has also been associated with human infections, causing diseases ranging in severity from ocular infections to fatal outcomes [51].

## 3. Conclusions

In conclusion, using our LC–MS/MS workflow for developing an in-house database for the dereplication of selected phytotoxins, we detected and reported the presence of putaminoxin in *C. kikuchii* and *C. sojina*, papyracillic acid and isosclerone in *D. longicolla*, and scytalone in *S. glycines*, *P. teres*, and *P. tritici-repentis* for the first time. Although cercosporin production had previously been reported in *C. nicotianae* from tobacco, the present study provides the first evidence of this phototoxin in *C. nicotianae* isolated from soybean, thereby expanding its known host range. Future studies will focus on developing a quantification method for these metabolites. These findings represent a preliminary chemical and biological assessment of the selected fungal isolates and underscore the importance of extraction protocols for the recovery of specialized metabolites. Extraction solvent, analytical methods, and culture conditions directly influence the chemical composition of the resulting extracts. Optimization of extraction parameters is therefore essential to maximize recovery of bioactive compounds. Equally important are the cultural conditions under which fungi are grown, as they profoundly modulate the expression of biosynthetic gene clusters. These environmental variables determine the metabolite profile and thus the accessible chemical space. Many fungal metabolites are produced only under specific growth conditions, whereas others remain cryptic unless activated by environmental cues. Given this complexity, future studies should adopt systematic approaches that vary culture media and growth conditions to unlock the full metabolic potential of these fungi. To complement these efforts, non-targeted metabolomics using mass spectrometry will be employed to capture the broadest possible spectrum of metabolites, including those already visualized in the preliminary TLC profiles. This will involve high-resolution LC–MS/MS analysis, coupled with advanced chemoinformatics workflows for peak detection, feature alignment, and deconvolution, as well as molecular networking prioritizing novel or unexplored compounds for downstream isolation and structural elucidation. Subsequent multivariate statistical analyses will enable comparisons across different culture conditions and extraction protocols, highlighting metabolites that vary significantly in abundance or appear uniquely in specific environments. This strategy will not only expand the accessible chemical space of these fungal species but also provide deeper insights into their ecological interactions, paving the way to the discovery of potential novel specialized metabolites, which can provide deeper insights into the chemical ecology of phytopathogenic fungi and clarify the potential roles of their metabolites in the onset and progression of plant diseases. Ideally, such efforts will be coupled with non-targeted metabolomics, which can provide deeper insights into the chemical ecology of phytopathogenic fungi and clarify the potential roles of their metabolites in the onset and progression of plant diseases.

## 4. Materials and Methods

### 4.1. General Experimental Procedure

Analytical TLC were performed on silica gel (Kieselgel 60, F_254_, 0.25) and the spots were visualized by exposure to UV radiation (253 nm), or by spraying first with 10% H_2_SO_4_ in MeOH and then with 5% phosphomolybdic acid in EtOH, followed by heating at 110 °C for 10 min. All these reagents were purchased from Sigma Aldrich (Darmstadt, Germany. LC-MS grade MeOH, formic acid, and quinaldic acid, which is used as an internal standard, were purchased from Sigma Aldrich (Darmstadt, Germany). The phenolic acids and alcohols Standard Mixture—V2 and the flavonoids standard mixture V2, purchased from MetaSci library (https://www.metasci.ca/, accessed on 12 September 2025), were used for peak identification, MRM method development and making calibration curves for quantification. The MeOH and EtOH are both 99.8% while EtOAc is 99.7%, all HPLC gradient grade. All these reagents were purchased from Sigma Aldrich (Darmstadt, Germany).

### 4.2. Fungi

The phytopathogenic fungi used in this study (Table 1) were isolated from commercial soybean, wheat, and barley crops across different regions of Argentina and are maintained in the fungal culture collection of the Plant Pathology Department, Faculty of Agronomy, University of Buenos Aires (Buenos Aires, Argentina). These isolates correspond to well-known plant pathogens and have been routinely handled under standard culture and maintenance procedures to preserve their aggressiveness and virulence. Their identification was previously carried out and published using both morphological traits and molecular methods [9,10,11,52,53,54,55,56]. Isolation originally followed conventional plant pathology procedures: plant tissues were surface-sterilized and incubated to promote fungal sporulation, after which single conidia were transferred directly or obtained through serial dilutions and plated onto culture media, yielding monosporic colonies for each pathogen. These isolates have been repeatedly used in multiple publications from our group, and several of the species (e.g., *C. kikuchii*, *C. nicotianae*, *P. teres*, *P. tritici-repentis*) have already been reported as resistant to different fungicide modes of action, while others (*S. glycines*, *C. sojina*, *Diaporthe* spp.) are currently under investigation for potential resistance [10,11,12,13].

### 4.3. Fungal In Vitro Growth

The fungal isolates used in this study were maintained on potato-dextrose-agar (PDA, Sigma-Aldrich Chemic GmbH, Buchs, Switzerland) plates and grown at 25 °C under near UV lights for 2 weeks. Small fragments of mycelia were used for seeding Roux 1-L bottles containing 200 mL of a sterile mineral defined liquid medium namely modified M1-D (several different salts plus saccharose 20.00 mg/L) [57]. Bottles were kept in still conditions at 25 °C in the dark in an incubator for 4 weeks according to literature on the study of toxin production [58] The suspension was then filtered by Whatman no. 4 filter paper, assayed for phytotoxic activity, and lyophilized for further purification.

### 4.4. Extraction of Specialized Metabolites from Fungal Liquid Culture Filtrated

A general method was used for the extraction of lipophilic metabolites from the fungal culture filtrates: the lyophilized culture filtrates were re-dissolved in distilled water (1/10 of initial volume about 150 mL) and extracted with EtOAc (3 × 150 mL). The organic extracts were combined, dried (Na_2_SO_4_), and evaporated under reduced pressure obtaining an oily or solid residue as reported in Table 1. The residual aqueous phase was acidified with formic acid up to pH 2. This latter was extracted with EtOAc (3 × 100 mL), and the organic extracts combined, dried (Na_2_SO_4_), and evaporated under reduced pressure giving them all an oily residue, as reported in Table 1.

### 4.5. Phytotoxic Assays Tested on Tomato Leaf

Organic extracts were assayed by using a leaf puncture assay on mung bean (*Vigna* sp.), tomato cv Regina di Fasano (*Solanum lycopersicum* L.) and soybean (*Glycine max* Merr). Extracts were dissolved in MeOH and then diluted with distilled H_2_O (final concentration 2 µg/µL in methanol (4%) in H_2_O). Fully expanded leaves were detached from plants and placed in moist chambers, at 25 °C and under constant fluorescent light. Droplets (20 µL) of solution were applied on adaxial sides of leaves, having previously punctured the leaf with a sterile needle. Tomato leaves were used because they are highly sensitive to non-specific toxins. Symptom appearance was observed daily up to 4 days after droplet application. Each treatment was repeated at least three times. Droplets of distilled water and methanol 4% were applied on leaves as negative controls.

### 4.6. Standards of Fungal Specialized Metabolites

The following 23 specialized metabolites, which had been previously isolated from the selected fungal species and for which pure standards were available, were chosen for targeted analysis: scytalone, isosclerone, ascosalitoxin, ascosalipyrone, (*R*)-mellein, 4-hydroxy-mellein, 6-methoxymellein, pinolidoxin, terpestacin, papyraciclic acid, cytocalasin A, cytocalasin B, gliotoxin, pypyropene A, sphaeropsidin A, ophobolin A, fusaproliferin, fusicoccin, catylenol, fisherindoline, putaminoxin, seiricardin C, and cyclopaldic acid [42,59,60,61,62,63,64,65,66,67,68,69,70,71,72,73,74] (Table S1 in Supplementary Materials) Metabolite standards were prepared by diluting the 1 mg/mL of the stock solution into CH_2_Cl_2_/MeOH (50% *v*/*v*) to reach the concentrations of 1 μg/mL to perform the manual optimization of MS/MS parameters.

### 4.7. NMR Analysis

1D and 2D NMR spectra were acquired in CDCl3 on a Bruker Avance III HD 400 DRX 600 spectrometer (Bruker, Billerica, MA, USA) equipped with a three-channel inverse (TCI) CryoProbe. The same solvents were used as the internal standard.

### 4.8. LC-MS/MS Analysis

The LC-MS/MS platform consisted of a UHPLC (Nexera Series LC-40, Shimadzu, Kyoto, Japan) coupled to a hybrid triple quadrupole/linear ion trap tandem mass spectrometer (QTRAP 4500, AB Sciex, Framingham, MA, USA) equipped with a Turbo V ion source. Instrument control, data acquisition, and processing were performed using the associated Analyst 1.6 software. The LC separation was carried out on a C18 column Cortecs (4.6 mm × 50 mm, particle size 2.7 mm) from Waters (Milford, MA, USA). Elution was performed at a flow rate of 0.7 mL/min with water containing 0.1% (*v*/*v*) formic acid as eluent A and ACN (Acetonitrile Merck) containing 0.1% (*v*/*v*) as eluent B, employing a linear gradient from 95% to 5% A in 10 min, and hold the solvent concentration for 2 min. The injection duty cycle was 16 min, considering the column equilibration time. Q1 resolution was adjusted to 0.7–0.1 amu fwhm for MRM, referred to as the unit resolution. Q3 was also set to the unit resolution in MRM mode. MS analysis was carried out in positive ionization mode using an ion spray voltage of 5500 V. The nebulizer and the curtain gas flows were set at 35 psi using nitrogen. The Turbo V ion source was operated at 500 °C with the gas 1 and 2 flow (nitrogen) set at 50 psi. Two suitable MRM transitions were selected for the specialized metabolites. The compound-dependent parameters were optimized using the manual optimization protocol in tuning mode. The Declustering Potential (DP) was set to 45, Exit Potential (EP) was set to 9, while the Q1 mass, the Q3 transition, and the optimized parameters are reported in Supplementary Table S1.

## Figures and Tables

**Table 1:** Fungal isolates used in this study and yields of the corresponding organic extracts at the original culture filtrate pH and at pH 2 (acid extraction).

Fungal Name	Source	EtOAc Extract ^1^ (Original pH) mg	EtOAc Extract ^1^ (pH 2) mg
*Cercospora kikuchii*	soybean crop 2016 (−24.7823, −63.7352)	12.4	1.5
*Cercospora nicotianae*	soybean crop 2016 (−26.5116, −64.4988)	13.6	18.9
*Cercospora sojina*	soybean crop 2009 (−32.690518, −62.170683)	8.4 ^2^	18.8
*Diaporthe longicolla*	soybean crop 2018 (−33.870845, −60.675023)	67.4	50.4
*Septoria glycines*	soybean crop 2021 (−33.406699, −61.195419)	32.1	65.4
*Pyrenophora teres*	barley crop 2021 (−37.7990, −57.9496)	181.0	186.4
*Pyrenophora tritici-repentis*	wheat crop 2018 (−34.5874, −60.4825)	51.7 ^2^	-

^1^ The residual are all homogenous oils except that obtained from *C. nicotiana* that is a red solid. ^2^ Original pH of several culture filtrates was about 5, except for *C. sojina* and *P. tritici-repentis*, which pH were 9 and 2, respectively.

**Table 2:** Phytotoxic activity of organic extracts from fungal isolates used in this study.

Isolates	Phytotoxic Activity of EtOAc Extract (Original pH) ^1^	Phytotoxic Activity of EtOAc Extract (pH = 2) ^1^
*Cercospora kikuchii*	Moderately phytotoxic	-
*Cercospora nicotianae*	Low phytotoxic activity	Moderate phytotoxic activity
*Cercospora sojina*	Low phytotoxic activity	Low phytotoxic activity
*Diaporthe longicolla*	Low phytotoxic activity	Low phytotoxic activity
*Septoria glycines*	Low phytotoxic activity	No phytotoxic activity
*Pyrenophora teres*	High phytotoxic activity	High phytotoxic activity
*Pyrenophora tritici-repentis*	Moderately phytotoxic	-

^1^ Toxic effects on leaves are expressed in the following way: no phytotoxic activity: no symptoms expressed; low phytotoxic activity: necrosis around 2–3 mm in diameter; moderately phytotoxic activity: necrosis 4–5 mm; high phytotoxic activity: necrosis 6–8 mm or wider.

**Table 3:** Optimized Q1 mass, transitions, and parameters for MRM analysis of selected specialized fungal metabolites.

Metabolite	Precursor Ion (*m/z*)	Product Ion (*m/z*)	CE ^a^	CXP ^b^	RT ^c^ (min)
scytalone	195.2 [M + H]^+^	177.0	18	15	3.48
		149.0			
isosclerone	179.2 [M + H]^+^	161.0	18	15	4.13
		133.0			
ascosalitoxin	265.1 [M + H]^+^	246.9	14	20	8.22
		237.0			
ascosalipyrone	239.2 [M + H]^+^	155.0	14	20	5.98
		85.0			
(*R*)-mellein	179.9 [M + H]^+^	162.0	20	13	6.97
		134.0			
4-hydroxymellein	195.4 [M + H]^+^	177.0	15	20	4.07
		149.1			
6-methoxymellein	208.8 [M + H]^+^	191.0	22	16	7.23
		162.8			
pinolidoxin	338.7 [M + H]^+^	181.1	11	14	7.65
		209.0			
terpestacin	402.8 [M + H]^+^	384.7	12	14	7.87
		367.2			
Papyracillic acid	226.9 [M + H]^+^	208.7	9	15	4.59
		166.9			
cytochalasin A	477.9 [M + H]^+^	459.9	20	23	8.39
		277.8			
cytochalasin B	480.0 [M + H]^+^	462.2	22	21	7.06
		425.8			
gliotoxin	326.9 [M + H]^+^	278.7	10	16	3.86
		230.7			
pyripyropene A	583.9 [M + H]^+^	265.1	43	9	7.32
		445.8			
sphaeropsidin A	346.6 [M + H]^+^	328.8	13	20	8.56
		283.0			
ophiobolin A	401.3 [M + H]^+^	364.9	13	20	8.56
		267.1			
fusaproliferin	445.0 [M + H]^+^	367.3	15	19	8.97
		348.9			
fusicoccin	681.0 [M + H]^+^	612.9	11	15	7.67
		373.1			
cotylenol	351.4 [M + H]^+^	301.8	13	19	6.35
		283.9			
fisherindoline	510.7 [M + H]^+^	234.6	23	28	7.63
		492.6			
putaminoxin	213.1 [M + H]^+^	195.2	7	20	6.27
		96.8			
seiricaricardin C	238.8 [M + H]^+^	109.0	12	27	12
		95.1			
cyclopaldic acid	238.7 [M + H]^+^	192.8	17	15	5.98
		220.7			

^a^ Collision energy. ^b^ Collision exit potential. ^c^ Retention time.

**Table 4:** Dereplication of specialized fungal metabolites by UHPLC-QTrap.

Fungus	EtOAc/pH	Metabolite
*Cercospora kikuchii*	2	Putaminoxin
*Cercospora sojina*	9	Putaminoxin
*Cercospora sojina*	2	NA ^1^
*Diaporthe longicolla*	5	Papyracillic acid
*Diaporthe longicolla*	2	Isosclerone
*Septoria glycines*	5	Scytalone
*Septoria glycines*	2	NA
*Pyrenophora teres*	5	Scytalone
*Pyrenophora teres*		NA
*Pyrenophora tritici-repentis*	2	Scytalone

^1^ NA: None of the specialized fungal metabolites reported in Table S1 were detected.

## Data Availability

The original contributions presented in this study are included in this article and Supplementary Material. Further inquiries can be directed to the corresponding authors.

## Supplementary Materials

The following supporting information can be downloaded at https://www.mdpi.com/article/10.3390/toxins17090466/s1, Table S1: List of the 23 metabolites used as references in the LC-MS/MS analysis. Figure S1: ^1^H NMR spectrum of cercosporin; Figure S2: *ed*-HSQC spectrum of cercosporin; Figure S3: HMBC spectrum of cercosporin; Figure S4: HRESIMS spectrum of cercosporin; Figure S5: TIC of scheduled MRM of organic extract.
