# Supplementary material for: Metabolic and Phytotoxic Profile of Phytopathogens in Main Extensive Crops of Argentina
Source: Toxins (Basel). 2025 Sep 18;17(9):466. doi: 10.3390/toxins17090466 (PMC12474131; doi:10.3390/toxins17090466)
Supplement: Supplementary file 1 [file toxins-17-00466-s001.zip › toxins-3831936-supplementary.pdf]

Supplementary Materials

Metabolic and Phytotoxic Profile of Phytopathogens in Main Extensive Crops of Argentina

Content:

S1. **Table S1.** List of the 23 metabolites used as references in the LC-MS/MS analysis.

S2. **Figure S1.** ^{1}H NMR spectrum of cercosporin (**1**) (Bruker 600MHz, $CDCl_3$)

S3. **Figure S2.** *ed*-HSQC spectrum of cercosporin (**1**) (Bruker 600MHz, $CDCl_3$)

S4. **Figure S3.** HMBC spectrum of cercosporin (**1**) (Bruker 600MHz, $CDCl_3$)

S5. **Figure S4.** HRESIMS spectrum of cercosporin (**1**)

S6. **Figure S5.** TIC of scheduled MRM of organic extract of: (A) Cercospora kikuchii, pH 2; (B) Cercospora sojina pH 9; (C) Diaporthe longicolla, pH 5; (D) Diaporthe longicolla, pH 2; (E) Septoria glycines, pH 5; (F) Pyrenophora teres, pH 5, (G) Pyrenophora tritici-repentis, pH 2.

Table S1. List of the 23 metabolites used as references in the LC-MS/MS analysis

Name	Structure	Purity[1]	Fungal source	Literature
scytalone	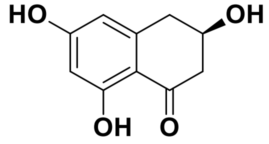	>95%		Evidente et al., 2000
isosclerone	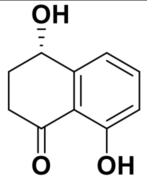	>95%		Evidente et al., 2000
ascosalitoxin	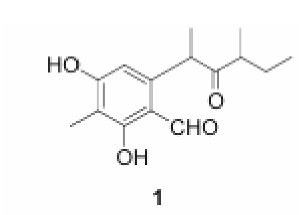	>95%		Evidente et al. 1983
ascosalipyrone	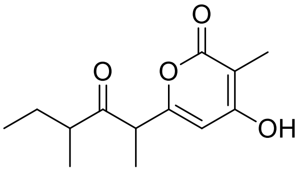	>95%	*Ascochyta fabae*	Barilli et al., 2023
(*R*)-mellein	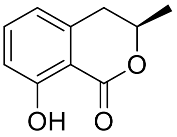	>95%	*Sphaeropsis sapinea*	Cabras et al., 2006
4-hydroxymellein	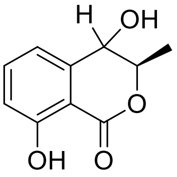	>95%	*Sphaeropsis sapinea*	Cabras et al., 2006
6-methoxymellein	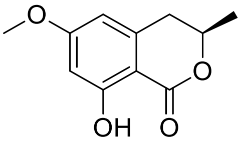	>95%	*Phoma chenopodiicola*	*Evidente M. et al., 2015*
pinolidoxin	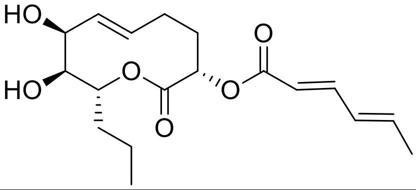	>95%	*Didymella pinodes*	Cimmino et al., 2012
terpestacin	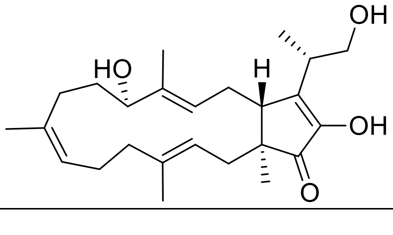	>95%	*Phoma exigua* var. *heteromorpha*	Masi et al., 2022

papyracillic acid	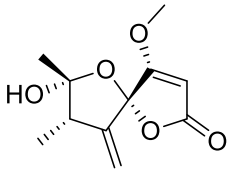	>95%	*Ascochyta agropirina* var. nana	*Evidente et al., 2009*
cytochalasin A	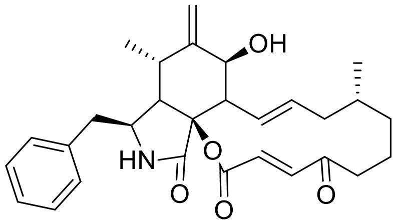	>95%	*Phoma exigua* var. *heteromorpha*	Capasso et al., 1987
cytochalasin B	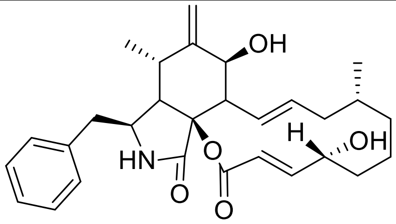	>95%	*Phoma exigua* var. *heteromorpha*	Capasso et al., 1987
gliotoxin	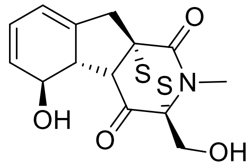	>95%	*Neosartorya pseudofichery*	Masi et al., 2013
pyripyropene A	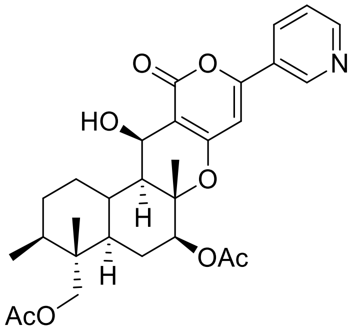	>95%	*Neosartorya pseudofichery*	Masi et al., 2013
sphaeropsidin A	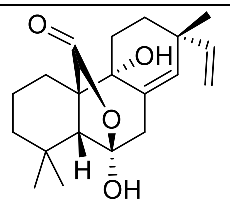	>95%	*Diplodia cupressi*	Evidente et al., 1996
ophiobolin A	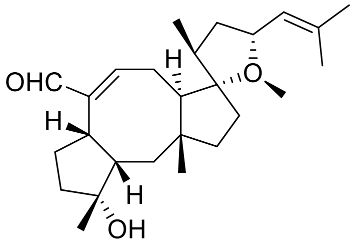	>95%	*Drechslera gigantea*	Evidente et al., 2006
fusaproliferin	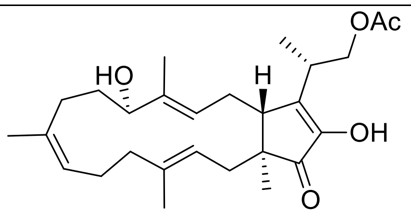	>95%	*Phoma exigua* var. *heteromorpha*	Masi et al., 2022

fusicoccin	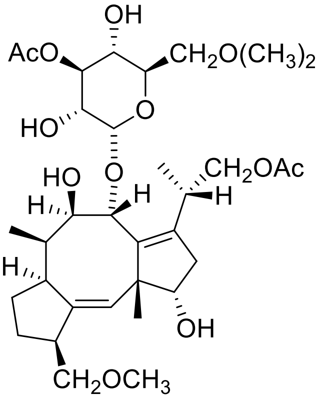	>95%	*Phomopsis amygdali*	Ballio et al., 1968
cotylenol	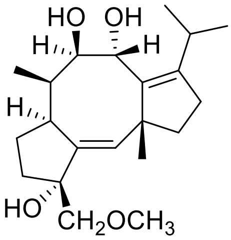	>95%	*Cladosporium* sp. 501–7W	Sassa 1972, Evidente et al. 2006
fisherindoline	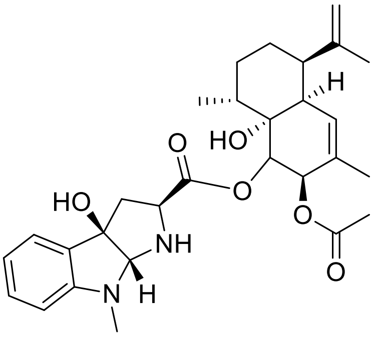	>95%	*Neosartorya pseudofichery*	Masi et al., 2013
putaminoxin	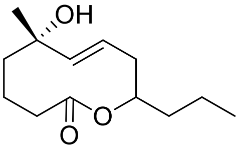	>95%	*Phoma putaminum*	Evidente et al., 1995
seiricardin C	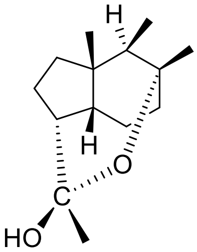	>95%	*Seiridium cardinale*	Evidente et al., 1993
cyclopaldic acid	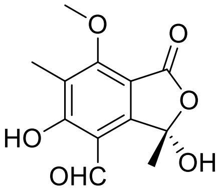	>95%	*Seiridium cupresii*	Graniti et al., 1992

[1]Determined by TLC and HPLC-ESI MS analysis.

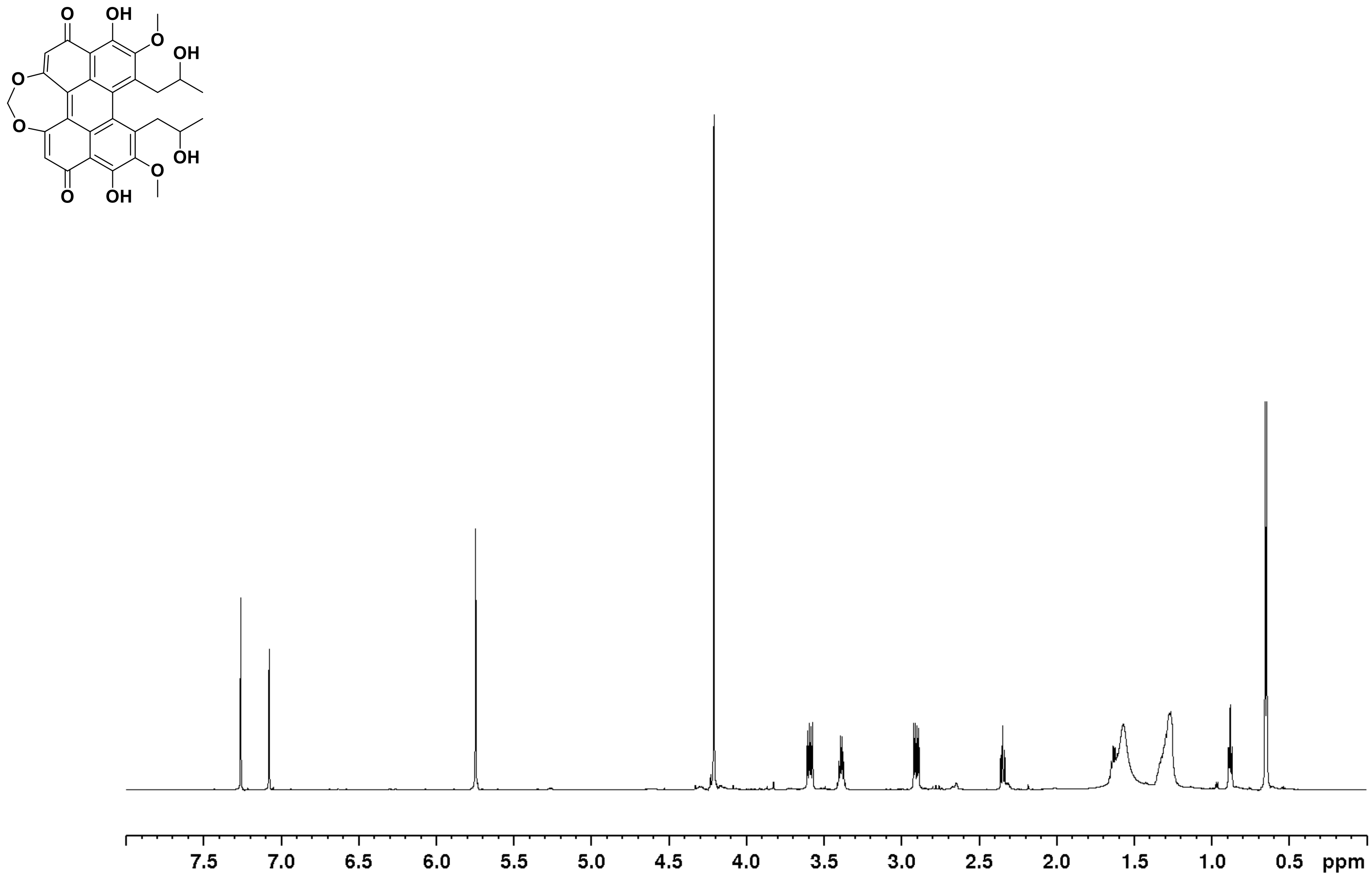

Figure S1. ^{1}H NMR spectrum of cercosporin (**1**) (Bruker 600MHz, $CDCl_3$)

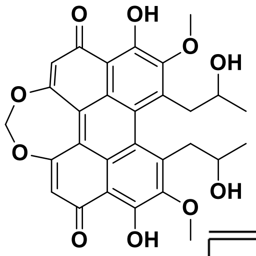

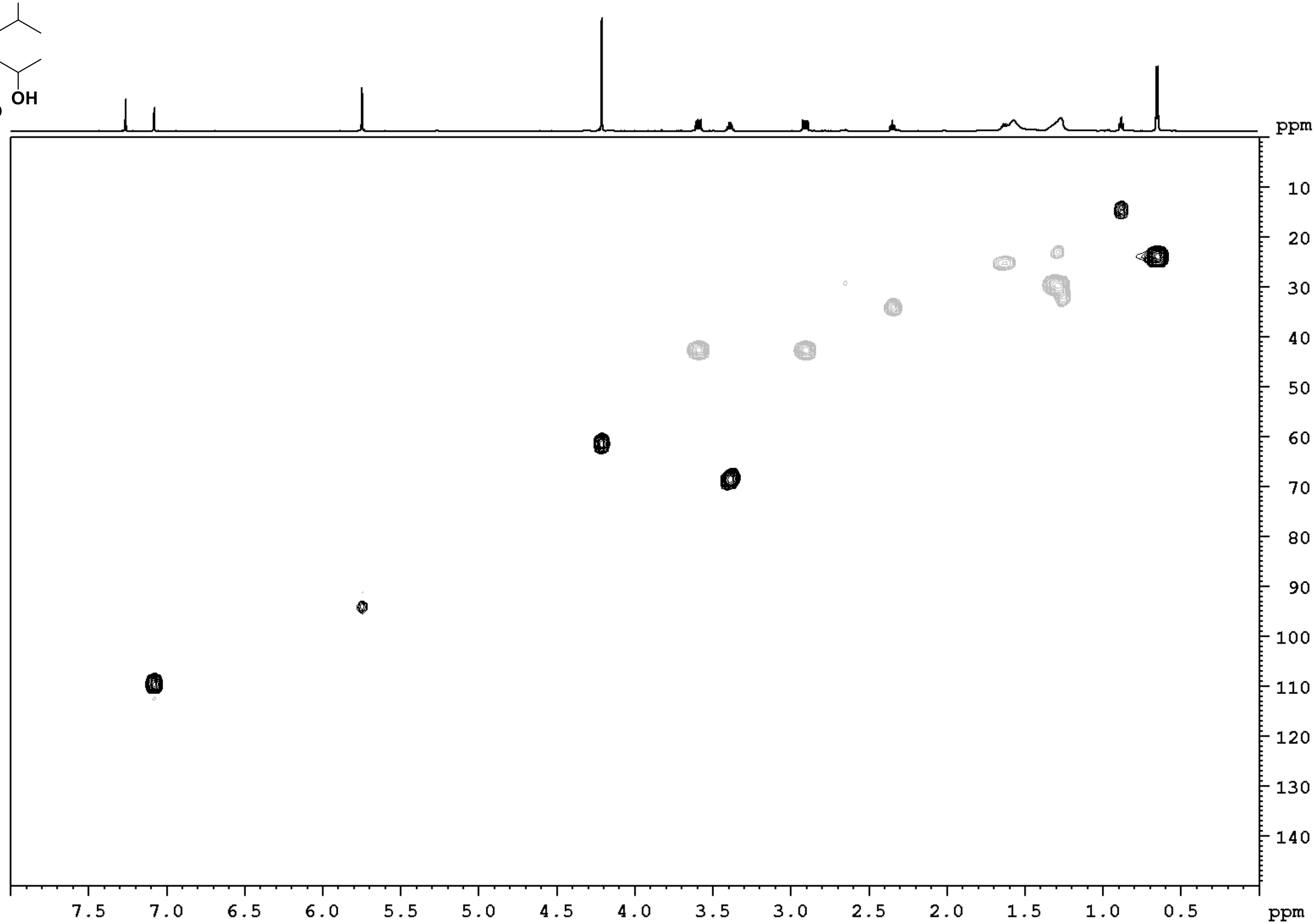

Figure S2. *ed*-HSQC spectrum of cercosporin (**1**) (Bruker 600MHz, $CDCl_3$)

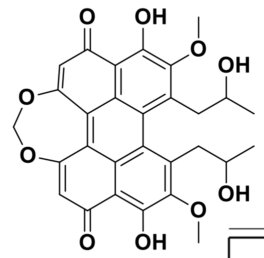

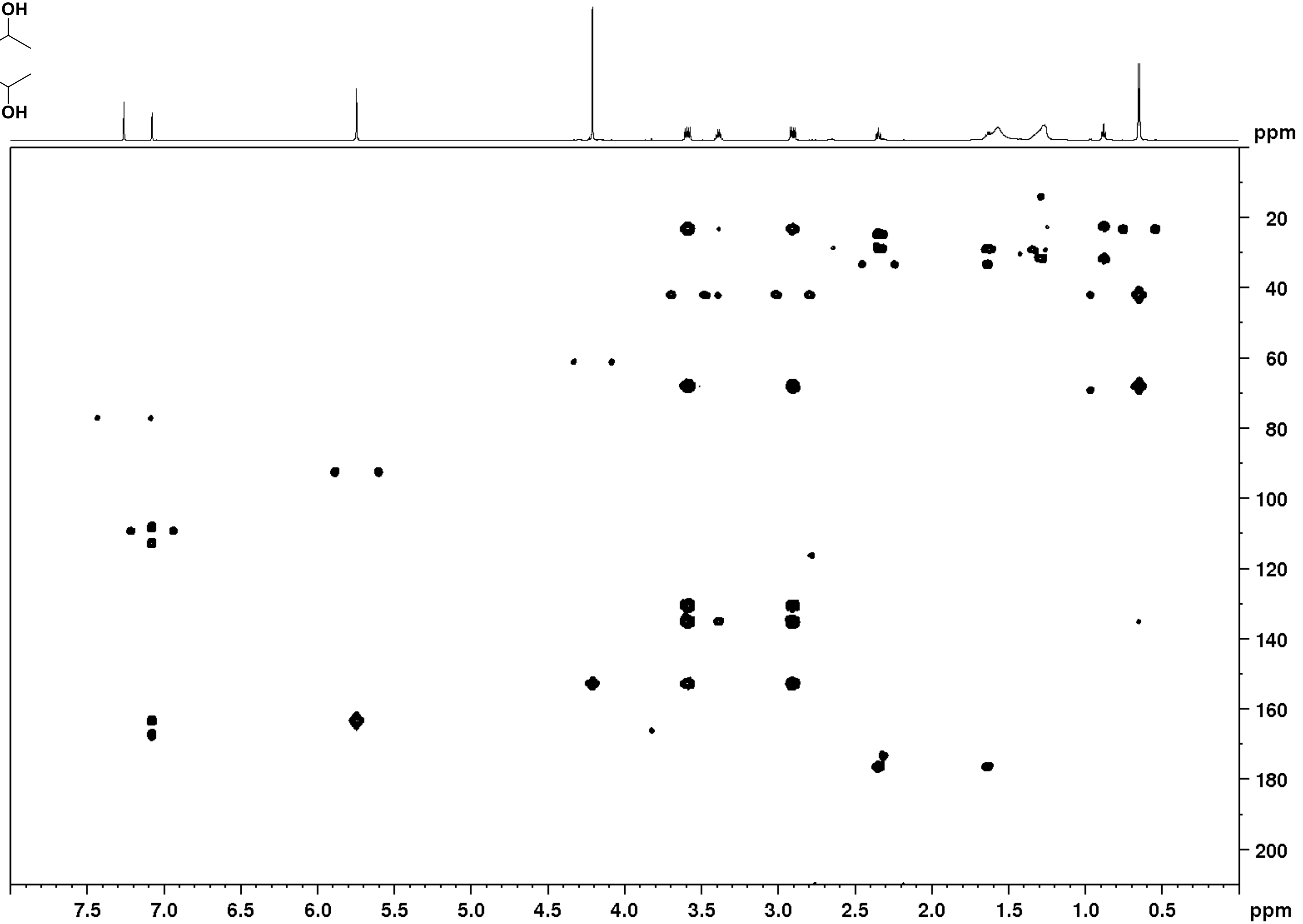

Figure S3. HMBC spectrum of cercosporin (**1**) (Bruker 600MHz, $CDCl_3$)

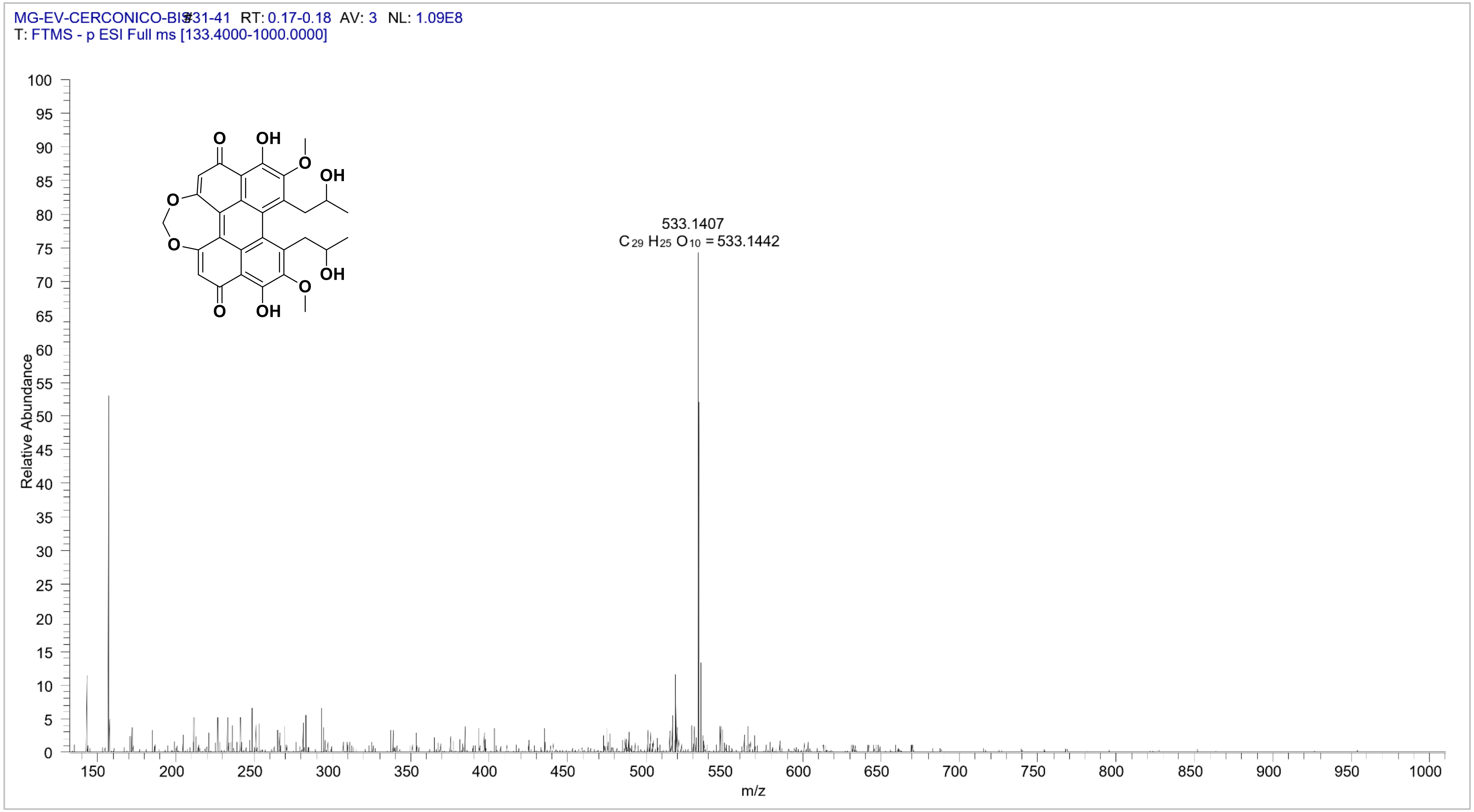

Figure S4. HRESIMS spectrum of cercosporin (**1**)

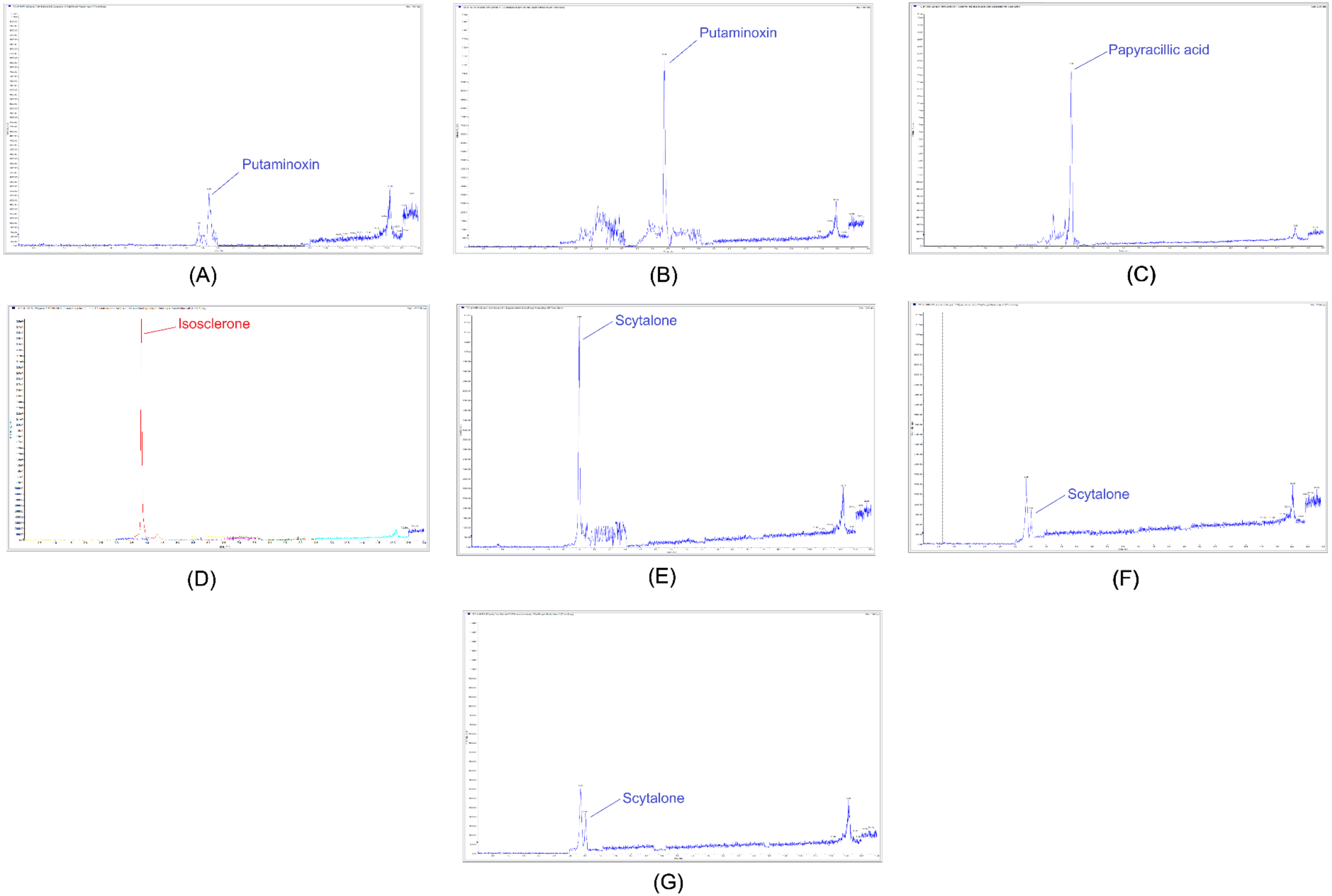

Figure S5. TIC of scheduled MRM of organic extract of: (A) *Cercospora kikuchii*, pH 2; (B) *Cercospora sojina* pH 9; (C) *Diaporthe longicolla*, pH 5; (D) *Diaporthe longicolla*, pH 2; (E) *Septoria glycines*, pH 5; (F) *Pyrenophora teres*, pH 5, (G) Pyrenophora tritici-repentis, pH 2.
